# Individualised prediction of psychosis in individuals meeting at-risk mental state (ARMS) criteria: protocol for a systematic review of clinical prediction models

**DOI:** 10.1186/s41512-019-0066-5

**Published:** 2019-11-21

**Authors:** Laura J. Bonnett, Filippo Varese, Catrin Tudur Smith, Allan Flores, Alison R. Yung

**Affiliations:** 1 Department of Biostatistics, University of Liverpool, Liverpool, UK; 2 School of Health Sciences, Division of Psychology & Mental Health, University of Manchester, Manchester, UK; 3 Greater Manchester Mental Health NHS Foundation Trust, Manchester, UK; 4 Centre for Youth Mental Health, University of Melbourne, Melbourne, Australia

**Keywords:** Prediction, Risk factors, ARMS, Psychosis, Meta-analysis

## Abstract

**Background:**

Psychotic disorders affect about 3% of the population worldwide and are associated with high personal, social and economic costs. They tend to have their first onset in adolescence. Increasing emphasis has been placed on early intervention to detect illness and minimise disability. In the late 1990s, criteria were developed to identify individuals at high risk for psychotic disorder. These are known as the at-risk mental state (ARMS) criteria. While ARMS individuals have a risk of psychosis much greater than the general population, most individuals meeting the ARMS criteria will not develop psychosis. Despite this, the National Institute for Health and Care Excellence recommends cognitive behavioural therapy (CBT) for all ARMS people.

Clinical prediction models that combine multiple patient characteristics to predict individual outcome risk may facilitate identification of patients who would benefit from CBT and conversely those that would benefit from less costly and less intensive regular mental state monitoring. The study will systematically review the evidence on clinical prediction models aimed at making individualised predictions for the transition to psychosis.

**Methods:**

Database searches will be conducted on PsycINFO, Medline, EMBASE and CINAHL. Reference lists and subject experts will be utilised. No language restrictions will be placed on publications, but searches will be restricted to 1994 onwards, the initial year of the first prospective study using ARMS criteria. Studies of any design will be included if they examined, in ARMS patients, whether more than one factor in combination is associated with the risk of transition to psychosis. Study quality will be assessed using the prediction model risk of bias assessment tool (PROBAST). Clinical prediction models will be summarised qualitatively, and if tested in multiple validation studies, their predictive performance will be summarised using a random-effects meta-analysis model.

**Discussion:**

The results of the review will identify prediction models for the risk of transition to psychosis. These will be informative for clinicians currently treating ARMS patients and considering potential preventive interventions. The conclusions of the review will also inform the possible update and external validation of prediction models and clinical prediction rules to identify those at high or low risk of transition to psychosis.

**Trial registration:**

The review has been registered with PROSPERO (CRD42018108488).

## Background

Identification of individuals at high and imminent risk of developing a first episode of psychosis is possible through the use of the “At-risk mental state” (ARMS) criteria [1, 2], a set of criteria suggestive of high-risk for psychosis originally proposed by Yung and operationally defined as low-grade “psychotic-like symptoms” that cause distress [3]. Meta-analytic evidence indicates that about 15–22% of ARMS individuals develop psychosis within 12 months from ARMS assessment [4, 5]. Identification of ARMS individuals therefore presents the opportunity for early intervention to prevent the onset of psychosis. However, most individuals meeting the ARMS criteria will not develop psychosis. This means that some ARMS individuals might be receiving unnecessary treatment and that the health services may be using costly interventions (preventive interventions with a growing evidence base, e.g. cognitive behaviour therapy; CBT) in people who may not need it. Furthermore, growing calls for improving the routine clinical management of people with ARMS (e.g. the UK’s Early Intervention Access and Waiting Standards, which now require all early intervention in psychosis services to assess, monitor and manage ARMS individuals [6]) are likely to result in an additional strain on resources and an urgent need to develop better systems to identify ARMS individuals that might be at the highest risk of developing psychosis and might therefore particularly benefit from receiving evidence-based preventive interventions.

Alternative indicators for assessing risk of transitioning to psychosis do exist, such as basic symptoms [7]. Prediction of risk in this way is comparable, or in some cases slightly superior than predicting transition to psychosis using ARMS [4]. However, ARMS is the most widely used approach to define the psychosis prodrome worldwide and forms part of national clinical assessment in the UK [6]. Indeed, ARMS is a standard approach within the UK with the whole workforce being trained in the assessment of ARMS. Consequently, there are specific calls around prediction of first episode psychosis focussing specifically on ARMS [4, 8].

Prognostic factors identify groups of patients at the highest risk and thus inform treatment decision making, patient counselling and policies [9]. Clinical prediction models combine multiple prognostic factors to predict individual outcome risk for individuals. Research to better stratify ARMS patients according to levels of risk of psychosis could facilitate more efficient use of resources available to health services. For example, those predicted to be at highest risk could be offered CBT, while lower risk patients could be offered less costly and less intensive regular mental state monitoring. In this way, a clinical prediction tool that can be used in routine practice could lead to the development of more cost-effective pathways and management plans.

Two recent systematic reviews summarised existing prediction models for ARMS patients [10, 11]. Both systematic reviews were undertaken in 2017, and since then, many additional relevant prediction models have been published. Additionally, the Prediction Study Risk of Bias Assessment Tool (PROBAST) guidelines were published in 2019 [12]. These facilitate thorough assessment of risk of bias of prediction model studies. Therefore, this systematic review is both timely and necessary.

Our work aims to systematically review all the evidence for current prediction models for transition to psychosis in individuals meeting the ARMS criteria. The findings should inform clinical practice and patient care by identifying demographic and clinical characteristics that show consistent evidence of predictive value when adjusted for other prognostic factors and by summarising the current prediction models and their predictive performance. It will also inform further research of prognostic factors and predictive models in this clinical area, including the potential development of a new clinical prediction rule.

### Research aims

The aim of this systematic review is to identify and summarise prediction models and clinical decision rules predicting transition to (first episode of) psychosis at 12 months, irrespective of whether an individual received an intervention or not, in people who have undergone an ARMS assessment in any clinical setting. This systematic review will identify and summarise studies of any prospective or retrospective design which utilise multiple prognostic factors in combination to predict the individualised risk of transition.

## Methods

### Selection criteria

#### Study design

The review will include any prospective or retrospective studies (i.e. cohort studies as well as randomised controlled trials of preventive interventions), with participants meeting the ARMS criteria, which have developed, compared, or validated a prediction model, or clinical prediction rule based on a model, combining multiple prognostic factors to predict the risk of transition to psychosis.

#### Patient group

This review will include individuals meeting the ARMS criteria (also called ultra-high risk (UHR) or clinical high risk (CHR) criteria). These are defined as (1) attenuated psychotic symptoms, (2) full-blown intermittent psychotic symptoms and (3) genetic/familial risk for schizophrenia in conjunction with a significant decrease in functioning and operationalised using suitable measures such as the Comprehensive Assessment of At-Risk Mental States (CAARMS) [13] or the Structured Interview for Prodromal Syndromes (SIPS) [14]. Other prodromal signs/symptoms distinct from ARMS (e.g. basic symptoms) will not be included. Studies with mixed populations, including those outside of the remit, will be included provided that the appropriate data for our defined group of patients is extractable.

Eligible prediction models will include patients at risk of transitioning to psychosis, and thus recruited to the study at the time of the ARMS assessment. There are no groups of ARMS individual for which any clinical prediction model predicting transition to psychosis cannot be used.

#### Setting

Studies in any setting will be included.

#### Potential prediction models

Studies must report a clinical prediction model utilising multiple prognostic factors to predict the risk of transition to psychosis following confirmation that a patient meets the ARMS criteria.

### Primary and secondary outcomes of our review

The primary outcome for the review will be the predictive accuracy of prediction models in relation to psychosis transition at 12 months, defined using standard diagnostic classification systems (DSM-III, DSM-IV, DSM-5, ICD-10, ICD-11) or commonly used ARMS assessment schedules (e.g. CAARMS or SIPS). The first 12 months is the highest risk period for psychosis onset [15] and the time when individuals are most distressed [16] and most likely to engage with services.

Secondary outcomes will be their predictive accuracy in relation to transition at other time points, quality of the developed models in terms of use of appropriate statistical methodology and the feasibility of using the model in clinical practice.

### Search strategy

Database searches will be conducted on PsycINFO, Medline, EMBASE and CINAHL. No restriction will be placed on the language of publication, but the searches will be restricted to 1994 onward, the initial year of the first prospective study using ARMS criteria [17]. No language restriction will be placed on the searches. Searches will use index terms and text words that encompass the patient group supplemented by terms relating to transition and prognostic factors (see sample Medline search in Appendix).

Database searches will be supplemented by:

1. Inspection of studies included in previous systematic reviews and meta-analyses of psychosis transition studies;
2. Inspection of reference lists of psychosis transition studies identified through the database searches;
3. Inspection of citations of psychosis transition studies identified through the database searches.

### Study selection

Titles and abstracts will be screened for relevance by two reviewers independently using pre-defined screening criteria. The screening criteria are broad and consider whether studies included patients meeting the ARMS criteria and developed or examined prediction models in relation to the transition to psychosis. Full texts of any potentially relevant articles will then be obtained, and two reviewers will independently assess the studies against the full inclusion criteria. When required, additional information to ascertain eligibility will be requested from study authors. Discrepancies in selection decisions will be discussed, and arbitration by another member of the research team sought to resolve such discrepancies.

Non-English studies will be translated where necessary to facilitate interpretation and data extraction. The study selection process will be documented using the PRISMA flow diagram [18]. EndNote reference management software will be used to record reviewer decisions, including reasons for exclusion [19].

### Data extraction

Data will be extracted by two reviewers independently using an in-depth piloted data extraction form. Disagreements will be resolved through discussion or referral to a third reviewer. The data extraction form will be pilot tested using a representative 5% sample of the studies to be reviewed. Consensus between review authors will be gained before any modifications are made to the form. If major changes are needed after the first testing, the pilot testing will be repeated on a new set of 5% of the studies. Data extraction will consider study characteristics, study design characteristics, patient characteristics, candidate prognostic factors considered including information on missing data, outcome measures, statistical methods employed and how prognostic factors included in the analysis were handled, and prediction model information. Data extraction specifically related to clinical prediction models will include the final model (its specification, included factors, values of regression coefficients and standard errors), how it was developed and any internal or external validation performance statistics for discrimination (such as the c-statistics or area under the curve) or for calibration (such as the expected/observed events ratio), together with their associated measures of spread. This will be informed by the Critical Appraisal and Data Extraction for Systematic Reviews of Prediction Modelling Studies (CHARMS) checklist which helps frame the review question, design the review and extract the relevant items from the reports of the primary prediction modelling studies [20]. 

### Assessment of study quality

The risk of bias (quality) of any studies developing or evaluating a prediction model will be assessed using the criteria described by Altman [21] and by PROBAST [12]. PROBAST involves assessment of participants, predictors, outcome and analysis.

### Evidence synthesis

Any studies reporting the development of a prediction model will be summarised narratively, in particular what prognostic factors were included in the final model, how the included variables were coded, what the specification of the model was and how it produces an individual outcome probability or risk score, the reported predictive accuracy of the model and whether the model was validated internally and/or externally, and if so how.

If multiple studies are found that externally validate the same prediction model, then calibration statistics (such as expected/observed events) and discriminatory statistics (such as the c-statistic or area under the curve) will be synthesised using random-effects meta-analysis methodology of Debray and Snell [22, 23] to summarise the model’s average performance across different settings and its predicted performance in a future setting. If there are updated versions of the same prediction model identified in our review, then only statistics for the most recent model will be included in the meta-analysis.

If we identify multiple prediction models that have been adequately externally validated, we will compare their performance narratively, taking into account the different case mix, how this relates to our own setting and also the quality of studies.

### Analysis of subgroups or subsets

If there are sufficient relevant prediction models available, subgroup analyses will synthesise calibration and discrimination statistics for studies conducted in different settings (countries) or different types of studies (prospective studies vs randomised studies vs randomised trials) or different model types (logistic vs survival analysis).

## Discussion

The results of our systematic review of existing studies have the potential to inform clinical management of patients diagnosed as ARMS. In particular, the results of the review will identify clinical prediction models for the risk of transition to psychosis after diagnosis. These will be informative for clinicians currently treating patients and considering whether to prescribe preventive interventions for psychosis or not for particular individuals. The review will also identify areas where the evidence for or against particular candidate prediction models is lacking, and this will lead to recommendations for initiating additional prediction model development and validation.

The models identified by this review as being potentially informative when estimating risk of transition to psychosis will be considered in related research by the authors (UK National Institute for Health Research HTA Project 17/31/05). This related project aims to update (via recalibration and extension to include new predictors as necessary) and externally validate any relevant existing prediction models or clinical prediction rules to identify a sub-group of patients at low risk of transition (in whom it is considered safe to undergo regular mental state monitoring) and in contrast to identify a sub-group of patients at high risk of transition to psychosis. Therefore, our systematic review is a crucial step towards the evidence-based use of prognostic factors and risk prediction in patients with ARMS considering treatment.

## Data Availability

Not applicable

## Appendix

Medline search strategy:

1. (ARMS or at risk mental state).ti,ab.
2. Basic symptoms.ti,ab.
3. Prodromal psychosis.ti,ab.
4. Psychosis risk.ti,ab.
5. (UHR or ultra high risk or CHR or clinical high risk).ti,ab.
6. Prodrom*.ti,ab.
7. Psychosis*.ti,ab.
8. 6 and 7
9. 1 and 7
10. 5 and 7
11. 1 or 2 or 3 or 4 or 5 or 8 or 9 or 10
12. (transition or transition$).ti,ab.
13. exp "Predictive Value of Tests"/
14. predict$.ti,ab.
15. exp Risk/
16. risk$.ti,ab.
17. prognos$.ti,ab.
18. or/13-17
19. 11 and 12 and 18

## Abbreviations

ARMS: At-risk mental status
CBT: Cognitive behavioural therapy
